# Humanized Mouse Model of Ovarian Cancer Recapitulates Patient Solid Tumor Progression, Ascites Formation, and Metastasis

**DOI:** 10.1371/journal.pone.0024420

**Published:** 2011-09-15

**Authors:** Richard B. Bankert, Sathy V. Balu-Iyer, Kunle Odunsi, Leonard D. Shultz, Raymond J. Kelleher, Jennifer L. Barnas, Michelle Simpson-Abelson, Robert Parsons, Sandra J. Yokota

**Affiliations:** 1 Department of Microbiology and Immunology, The State University of New York, University at Buffalo, Buffalo, New York, United States of America; 2 Pharmaceutical Sciences, The State University of New York, University at Buffalo, Amherst, New York, United States of America; 3 Department of Gynecologic Oncology, Roswell Park Cancer Institute, Buffalo, New York, United States of America; 4 Jackson Laboratory, Bar Harbor, Maine, United States of America; Cedars-Sinai Medical Center, United States of America

## Abstract

Ovarian cancer is the most common cause of death from gynecological cancer. Understanding the biology of this disease, particularly how tumor-associated lymphocytes and fibroblasts contribute to the progression and metastasis of the tumor, has been impeded by the lack of a suitable tumor xenograft model. We report a simple and reproducible system in which the tumor and tumor stroma are successfully engrafted into NOD-scid IL2Rγ^null^ (NSG) mice. This is achieved by injecting tumor cell aggregates derived from fresh ovarian tumor biopsy tissues (including tumor cells, and tumor-associated lymphocytes and fibroblasts) i.p. into NSG mice. Tumor progression in these mice closely parallels many of the events that are observed in ovarian cancer patients. Tumors establish in the omentum, ovaries, liver, spleen, uterus, and pancreas. Tumor growth is initially very slow and progressive within the peritoneal cavity with an ultimate development of tumor ascites, spontaneous metastasis to the lung, increasing serum and ascites levels of CA125, and the retention of tumor-associated human fibroblasts and lymphocytes that remain functional and responsive to cytokines for prolonged periods. With this model one will be able to determine how fibroblasts and lymphocytes within the tumor microenvironment may contribute to tumor growth and metastasis, and will make it possible to evaluate the efficacy of therapies that are designed to target these cells in the tumor stroma.

## Introduction

Both normal and neoplastic human tissues have been successfully engrafted into T cell and B cell-deficient *prkdc^scid^ (scid)* mice. The first successful engraftment of human cells into these C.B-17 *scid* mice was reported over 20 years ago [1]. The use of human tissue xenografts in these immunodeficient mice has since led to insights into the biology of human cancer, autoimmunity and infectious diseases [2]. The potential of using several different human tumor xenograft models to study and evaluate anti-cancer therapies along with the models' limitations and pitfalls has been reviewed [3]. One of the greatest impediments of the earlier xenograft models was the host-versus-graft (HVG) response that was observed following the implantation of human tissues. While *scid* mice lacked functional B and T cells, these mice had an intact innate immune response that was responsible for the complex cellular and molecular HVG response. The intensity of this HVG response varied considerably from mouse to mouse and with the histological type of tumor used for engraftment [3]. Enhanced levels of human tissue engraftment have depended primarily upon genetic modifications of immunodeficient host mice [2]. A major breakthrough was the generation of immunodeficient mice that are homozygous for targeted mutations at the interleukin-2 receptor γ chain locus [2]. These mice are severely impaired in the development and function of T cells, B cells and NK cells [2]. One example of this new type of immunodeficient mouse strain is the NOD.Cg-*Prkdc^scid^IL-2rg^tm1Wjl^*, abbreviated NSG. Immunodeficient mice lacking the IL-2 receptor γ chain have been found to support the prolonged engraftment of human hematopoietic cells and peripheral blood mononuclear cells [2] better than previous immunodeficient mouse strains.

A xenograft model in which human tumors and autologous tumor-associated T and B cells could be co-engrafted and their interactions studied *in vivo* would provide an opportunity to determine how human immunocompetent cells and tumor cells influence each other. The findings could potentially be used to evaluate immunotherapeutic approaches to cancer. Further, in view of the increasing awareness that nonmalignant stromal cells including fibroblasts, epithelial cells and other leukocytes interact with and have an impact upon tumor growth and metastases [4], the development and use of models in which the tumor microenvironment is maintained in the xenograft is also critical to gaining insights into the pathogenesis of tumor progression. By implanting non-disrupted pieces of human tumors subcutaneously into NSG mice, xenografts were established in which the tissue architecture, including tumor-associated leukocytes, stromal fibroblasts and tumor cells was preserved and maintained for prolonged periods [5]. This model proved useful in demonstrating the ability of exogenous IL-12 delivered by biodegradable microspheres to activate quiescent memory T cells within the tumor microenvironment [5]. However, because these tumor xenografts were not established orthotopically, and only limited evidence of tumor spreading was observed, the model did not accurately reflect the patterns of growth and metastasis that are observed in cancer patients, and was therefore of limited potential value in identifying factors that contribute to tumor metastasis or for assessing the efficacy of immunotherapeutic protocols.

A tumor xenograft model has been developed and is reported here in which human epithelial ovarian tumor xenografts are established orthotopically, and the pattern of tumor growth and metastasis reflects that which is observed in ovarian cancer patients. Tumor-associated T and B lymphocytes and fibroblasts co-engraft within tumor nodules and the T cells remain functional and responsive to exogenously administered cytokine. CA125 is present in the sera and ascites of CA125+ tumor-bearing mice and provides an opportunity to monitor the presence and progress of tumor xenografts periodically. It is expected that this model will make it possible to study the ability of human tumor-associated leukocytes and tumor stroma to modulate *in situ* tumor progression. Moreover, the model provides the potential to evaluate the efficacy of single, as well as combination, therapeutic approaches to ovarian cancer.

## Results

### Human ovarian epithelial tumors engraft orthotopically in the ovary and other organ sites following the intraperitoneal (i.p.) injection of tumor-derived cell aggregates into NSG mice

It was established previously that the subcutaneous implantation of solid pieces of fresh human tumor tissues into NSG mice resulted in the establishment of tumor microenvironments [5]. The advantages of this approach over previous methods were that the resultant xenografts maintained their original architecture, including tumor-associated leukocytes, stromal fibroblast and tumor cells, and the xenografts survived for prolonged periods without HVG interference or infiltration of host cells. A major limitation of this earlier xenograft model was that it failed to reflect the patterns of tumor growth and spreading that are observed in cancer patients, and some tumors failed to engraft or resulted in xenografts with large areas of necrosis due to an inadequate vascularization of the solid pieces of tumors.

**Figure 1:**
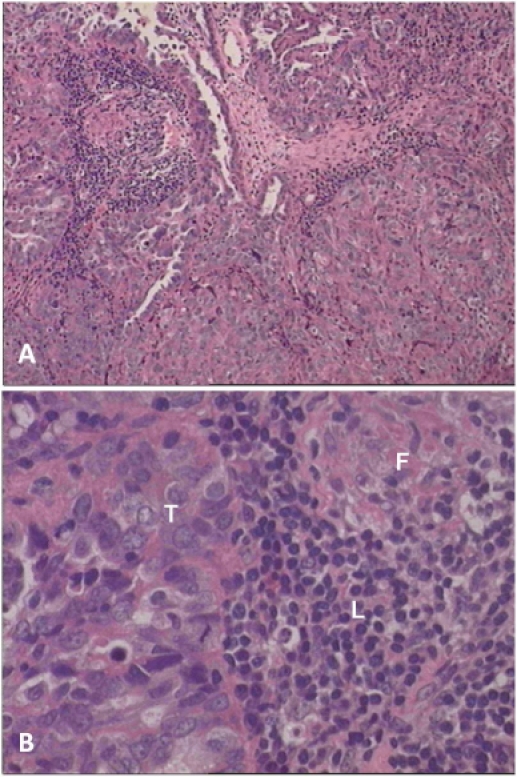
Histology of the original tumor. A section of a papillary serous adenocarcinoma of the ovary stained with hemotoxylin and eosin (H&E). Shown in A 100× magnification and in B 400× magnification clusters of tumor cells (T) that show lymphocytic infiltration (L), along with fibrous connective tissue (F).

**Figure 2:**
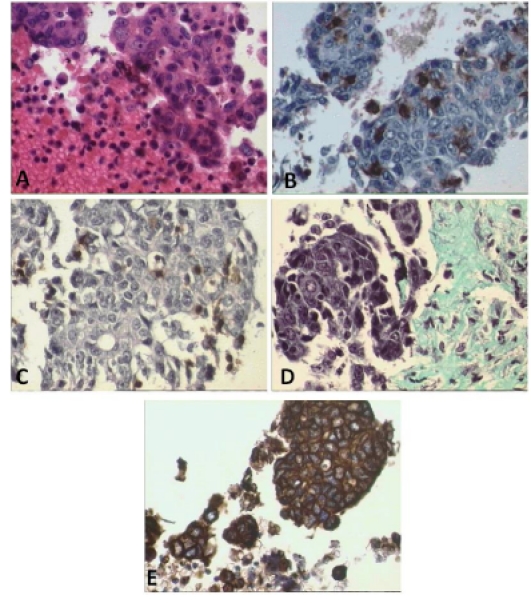
Presence of tumor, T cells and fibroblasts in tumor cell aggregates. Histology and immunohistochemistry of tumor-derived cell aggregates derived by mild disruption of a primary ovarian tumor. H&E staining show clusters of cells of different sizes (A) Immunohistochemical staining for human CD45 (B) and CD3 (C) shows evidence of human leukocytes and T cells, i.e. dark brown stained cells. Trichrome staining (D) reveals the presence of collagen which is produced by fibroblasts and stains aquamarine. Tumor cells stain dark brown with immunohistochemical stain for cytokeratin (E). All figures are at 400× magnification.

We report here a method of engraftment, in which ovarian tumors can be successfully established as xenografts orthotopically in the ovary and other organ sites of recipient mice. The tumor growth reflects the pattern and progression observed in ovarian cancer patients. This has been accomplished by injecting a suspension of cell aggregates (that is derived from a mild disruption of solid ovarian tumors) i.p. into NSG mice (see Methods section). To insure successful tumor engraftment, the tumor biopsy tissues must consist of areas of viable tumor cells that include tumor-associated lymphocytes, and fibroblasts as shown in Fig. 1. The tumor-derived-cell suspensions resulting from the disruption of the fresh solid tumor tissues contain small (200–300 µm diameter) aggregates of cells that include CD45+ leukocytes, cytokeratin positive tumor cells, CD3+ T cells and trichrome positive collagen which is produced by fibroblasts (Fig. 2A–E). Cell aggregates derived from ovarian tumors obtained from five different patients (Experiment 1–5) were injected i.p. into NSG mice and the recipient mice sacrificed at the times indicated in Table 1. While histological evidence of the attachment and growth of the tumor cell aggregates were observed on the omentum as early as 8 days post i.p. inoculation (data not shown) gross and histological evidence of tumor and tumor stroma within different organ sites (discussed below) was not established until 80 to 177 days post inoculation (Table 1). None of the mice showed any obvious clinical signs of tumor development during the first 10 weeks of observation following the tumor inoculation. Death was often the first sign of tumor engraftment. One mouse from each experiment died without evaluation, and was not included in the final analysis.

**Table 1:** Frequency of Tumor Development Following i.p. Injection of Tumor Cell Aggregates Derived from Ovarian Cancer Patient Solid Tumors.

Experiment #^a^	Tumor Type	# Mice Implanted^b^	Days Post Inoculation	Evidence of Tumor^c^
**1**	Papillary Serous Carcinoma	10	80	8/9
**2**	Papillary Serous Carcinoma	10	82	9/9
**3**	Papillary Serous Carcinoma	10	115	9/9
**4**	Papillary Serous Carcinoma	10	140	8/9
**5A**	Adenocarcinoma	15	116	13/15
**5B**	Adenocarcinoma	5	177	4/4

a Each experiment was conducted with tumor tissue derived from a different ovarian cancer patient. In experiment 5A and B the cell aggregates was generated from the same tumor.

b Mice were injected i.p. with tumor-derived cell aggregates obtained from five different ovarian cancer patients, that is generated as indicated in Materials and Methods section.

c Evidence of tumor was established by the presence of grossly detected tumor nodules in the peritoneal cavity, histological evidence of tumor in the organs or both. In each experiment, #1–4, one of the 10 mice died without evaluation. In experiment #5 one of the mice was sacrificed at day 85 post inoculation without any evidence of gross tumor. In experiment 5B – one of the mice died without evaluation.

**Figure 3:**
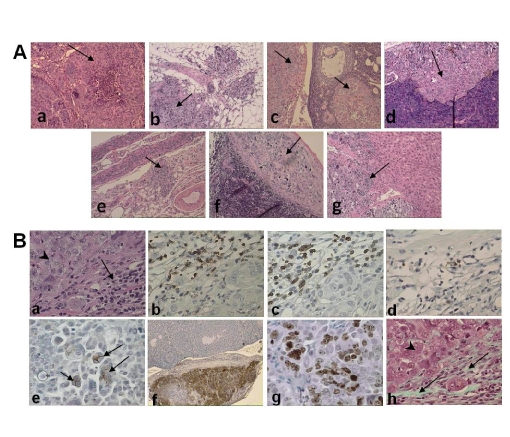
Tumor and stroma in multiple organs in NSG mice following injection of tumor aggregates. H&E staining shows evidence of tumor (see arrows) in peritoneal nodule from omentum (Aa and Ab), ovary and periovarian fat (Ac), pancreas (Ad), uterus (Ae), spleen (Af), and liver (Ag). These tumors resulted from the i.p. injection of tumor-derived cell aggregates derived from a papillary serous carcinoma of the ovary. Mice were sacrificed 140 days post inoculation. Lymphocytes (arrow) were observed in juxtaposition with tumor cell (arrowhead) (Ba). Immunohistochemical staining (B) shows the presence of human CD45+ leukocytes (Bb), CD3+ T cells (Bc), CD20+ B cells (Bd), CD138+ plasma cells (Be), and HLA+ tumor nodule adjacent to the ovary (Bf). Proliferation of tumor cells is shown by positive stain with KI67 (Bg), and evidence of stromal fibroblasts illustrated by trichrome staining of collagen see arrows (Bh). Arrow head shows tumor cells. All sections in A are at 100× magnification and in B the sections are at 400× magnification.

Nodules detected grossly within the peritoneal cavity of mice injected with tumor-derived cell aggregates were confirmed histologically to be tumors (Fig. 3Aa and 3Ab). The tumor nodules varied in diameter from 2 mm to 10 mm and were most frequently observed within or near the area of the omentum, i.e. adjacent to the stomach, spleen and pancreas. In some mice tumors were observed grossly on the surface of the spleen and liver. To determine whether the tumors had spread to other organ sites within the peritoneal cavity, sections of each organ were taken, fixed and stained. Fig. 3Ac–3Ag reveal the presence of tumor in the ovary, periovarian fat, pancreas, uterus, spleen and liver from a mouse sacrificed 140 days after the injection of tumor-derived cell aggreages (Experiment #4).

The frequency of tumors observed histologically in different organ sites varied from tumor to tumor and from mouse to mouse for each tumor-derived cell aggregate implanted. In a typical example of the organ distribution in 15 mice, 116 days after the i.p. injection of cell aggregates derived from solid ovarian tumors (Experiment 5A, Table 1), histological evidence of tumor was detected in the ovary of 10 mice, the spleen of 10 mice, the liver of 8 mice, and the uterus of 2 mice. It is considered likely that this organ site involvement is an underestimate of the actual frequency of tumors in the different organs, because only a small area of each organ was examined histologically.

### Human CD45+, CD3+, CD20+, CD138+ Leukocytes, Fibroblasts and Proliferating Tumor Cells are Present in the Microenvironment of Tumor Xenografts

Sections of tumor nodules removed from the peritoneal cavity and stained with hemotoxylin and eosin revealed areas in which lymphocytes (see arrow) were present in juxtaposition with tumor cells (see arrowhead) (Fig. 3Ba). Immunohistochemical staining of this tissue established that the tumor-associated lymphocytes were human CD45+ and included CD3+ T cells, CD20+ B cells, and CD138+ plasma cells (Fig. 3Bb–Be). Tumor cells stained positively for HLA Class I (Fig. 3Bf), and included actively dividing Ki67 positive cells (Fig. 3Bg).

A trichrome stain of the tumor nodules showed that in addition to the inflammatory cells, fibroblasts were present within the microenvironment of the tumor xenografts (see arrow) (Fig. 3Bh). The fibroblasts stained positively for HLA Class I (data not shown).

We conclude that the histological architecture and cellular composition including the tumor cells, inflammatory leukocytes and fibroblasts of the original tumor tissue are maintained for prolonged periods (up to 177 days post-engraftment) in the tumor xenograft, and that tumor cells continue to proliferate within the tumor nodules present in the peritoneal cavity.

### Tumor Ascites Development in NSG Mice Inoculated with Ovarian Tumor-Derived Cell Aggregates

Ovarian cancer patients typically develop a tumor ascites in the later stages of their disease [6], [7]. To determine whether tumor ascites develop in the tumor xenograft model, NSG mice were monitored for prolonged periods following the i.p. inoculation of tumor-derived cell aggregates. Distended abdomens were observed in mice by 14 weeks after tumor injection suggesting the presence of ascites. Paracentesis confirmed the presence of ascites fluid in the peritoneal cavity of the mice. To determine whether viable tumor cells were present within the ascites fluid, the ascites fluids from tumor bearing mice (generated by the i.p. inoculation of ovarian tumor-derived cell aggregates from three different ovarian cancer patients) were injected i.p. into naïve NSG mice. Eighty-seven to 94 days post-inoculation mice had histological evidence of tumor development in the ovary, uterus, liver, spleen and lung (data not shown). Most of these secondary recipients had also developed ascites by this time. These results establish both the presence of viable tumor cells in the ascites fluid of the original tumor bearing mice, and the ability to sub-passage the tumor and thereby expand the number of mice with tumor xenografts. Tumors have now been successfully sub-passaged three times. After the third passage the tumor xenografts appear to be largely devoid of human tumor associated fibroblasts and tumor associated lymphocytes.

### Presence of CA125 in the Sera and Ascites of Ovarian Tumor Bearing NSG Mice

CA125 is a high molecular weight glycoprotein that is elevated in the serum of approximately 90% of patients with advanced epithelial ovarian cancer [8], [9], and is used to monitor tumor progression and response to chemotherapy in ovarian cancer patients [8]. Because the only clinical sign of tumor development in the mice was tumor ascites that occurred very late, it was of interest to determine whether CA125 levels could be detected in the NSG mice engrafted with ovarian tumor-derived cell aggregates, and to establish whether this marker could be utilized to periodically monitor the presence and progression of the human tumors in mice following the tumor inoculation. The cell aggregates were derived from solid tumors of three different ovarian cancer patients with elevated serum levels (>500 u/ml) of CA125. Each of the three tumor-derived cell aggregates was injected i.p. into 10 NSG mice and the CA125 levels in the serum and ascites were assayed at different times after tumor inoculation. By 80 days post tumor inoculation, CA125 was present in the ascites and sera of all mice inoculated with tumors derived from the three different patients. In one of the three groups of mice the animals were bled periodically and the serum levels of CA125 determined. The CA125 in the serum increased with time reaching a level of greater than 500 units/ml 100 days post tumor-derived cell aggregate inoculation. The amount of CA125 in the ascites varied considerably from tumor to tumor reaching levels of 400–5800 units/ml 85–116 days post tumor-derived cell aggregate inoculation. The ability to detect CA125 in the serum and ascites of tumor xenograft bearing mice is reflective of yet another event that is observed in ovarian cancer patients, and is significant as it provides a way to periodically monitor the presence and progression of the tumor, and ultimately to assess the therapeutic efficacy of different treatment protocols.

### Metastatic Spread of Ovarian Tumor Xenografts from the Abdominal Cavity into the Pleural Space

**Figure 4:**
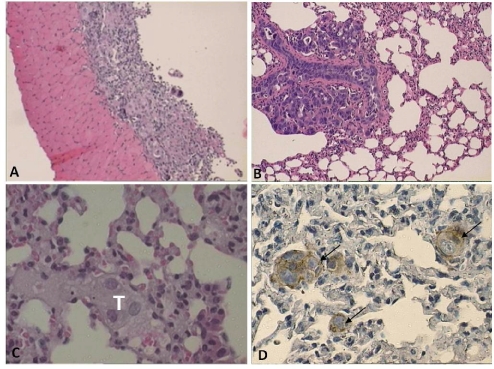
Spontaneous metastasis of tumor xenograft into the lung. H&E staining (100× magnification) reveals the presence of tumor in the diaphragm (A), and in the lung 100× magnification (B) and 400× magnification (C). The immunohistochemical staining of the tumor in the lung for HLA Class I (D) establishes that these tumors are of human origin. The sections were made from the tissues of a mouse 16 weeks after the i.p. injection of a suspension of tumor = derived cell aggregates.

The intra-abdominal spreading that we observed following the i.p. inoculation of ovarian tumor-derived cell aggregates into NSG mice, and the generation of tumor ascites fluid are consistent with what is seen in Stage IIIc ovarian cancer patients [7]. At this stage the disease may involve the omentum, bowel mesentery, abdominal peritoneal surfaces, serosal surface of the bowel and the diaphragm. Mice that had developed tumor ascites by 16 weeks after the i.p. injection of tumor-derived cell aggregates, had gross evidence of tumors on the serosal surfaces of the bowel and omentum and were carefully examined for diaphragmatic tumor implants. Gross evidence of possible tumor growth was observed on the diaphragm of 12 of 15 mice inoculated with the tumor-derived cell aggregates. Histological analysis confirmed the presence of tumor growth on the diaphragm (Fig. 4A).

Stage IV disease patients present with evidence of extra-abdominal spreading of the tumor and this may involve the pleural space with metastasis into the lung parenchyma [7]. While no gross evidence of tumor metastasis in mice was observed by 16 weeks post tumor-derived cell aggregate inoculation, histological analysis of the lungs established that micro-metastasis of the tumor from the peritoneal cavity had occurred in 4 of 15 mice. Small foci of tumors observed in the lung (Fig. 4B,C) stained positively for HLA Class I (Fig. 4D). The number of mice with lung metastasis is likely an underestimate as only a small portion of the lung was examined histologically.

Our collective findings indicate that the slow and progressive pattern of tumor growth and metastasis of ovarian tumors in NSG mice following the i.p. inoculation of fresh human ovarian tumor-derived cell aggregates closely reflects that seen in patients who develop into Stages IIIc and IV of disease which accounts for over 70% of the ovarian cancer patients seen in the clinic [7].

### Human T cells present in xenografts produce IFN-γ upon activation with exogenous IL-12 and plasma cells constitutively produce immunoglobulin (Ig)

Lymphocytes including effector memory CD4+ T cells [10], NY-ESO-1 tumor specific CD8+ T cells [11], CD4+ and CD8+ regulatory T cells [12]–[14], and TH17 cells [15] have been found within ovarian tumor microenvironments. These and other tumor infiltrating leukocytes have been shown to be associated with both the enhancement and inhibition of tumor progression [16]. It was of interest and relevant to the potential utility of our humanized mouse model to establish whether the tumor-associated lymphocytes that were found in the tumor xenografts remained viable and functional. Having established by immunohistochemistry the presence of human CD45+ leukocytes CD3+ T cells, CD20+ B cells and CD138+ plasma cells in the tumor microenvironment of xenografts established following the i.p. injection of tumor-derived cell aggregates (Fig. 3B), the viability and the capacity of these cells to respond to exogenous cytokine stimulation *in vivo* were investigated.

We have previously established that T cells in the tumor microenvironment have an attenuated TCR driven activation signal, but remain viable and respond to stimulation with IL-12 by producing IFN-γ [17], [18]. If the T cells present in the tumor xenografts remained viable we predicted that there would be a significant increase of IFN-γ in the sera of tumor bearing mice following the treatment of the mice with IL-12 [19]. Tumor bearing mice (21 days post tumor inoculation) were injected i.p. with either human IL-12 loaded liposomes (50 µg of IL-12 per mouse) or control (empty) liposomes, and their sera were assayed 5 days later for the presence of human IFN-γ. While the level of IFN-γ in the sera of the IL-12 treated mice varied from mouse to mouse, 9 out of 10 of these mice showed significantly elevated levels of IFN-γ (233–5392 µg/ml) (Table 2). All 5 of the tumor bearing mice treated with control liposomes had lower serum levels of IFN-γ ranging from 32–88 pg/ml. These results suggest that the human tumor-associated T lymphocytes, and possibly NK cells, present in the tumor bearing mice are both viable and responsive to IL-12. The single treatment with IL-12 did not result in a significant decrease in tumor progression compared to control mice. Future studies are planned to assess the effect of multiple treatments of the mice with IL-12 either alone or in combination with chemotherapy.

**Table 2:** Evidence of Viability and Function of T Cells and Plasma Cells in Tumor Bearing Mice.

Treatment of Mice^a^	IFN-γ (pg/ml)^b^	Human Ig (µg/ml)^c^
Empty Liposome	32.0	500
Empty Liposome	87.9	550
Empty Liposome	38.2	38
Empty Liposome	32.0	<25
Empty Liposome	32.0	<25
IL-12 Liposome	66.1	458
IL-12 Liposome	469.6	738
IL-12 Liposome	416.9	435
IL-12 Liposome	842.1	816
IL-12 Liposome	1379.1	456
IL-12 Liposome	5392.4	Died
IL-12 Liposome	357.9	1123
IL-12 Liposome	233.7	1361
IL-12 Liposome	376.5	868
IL-12 Liposome	3191.8	743

a NSG mice injected i.p. with control empty liposomes or liposomes loaded with IL-12. The dose of IL-12 was 50 µg/mouse. All mice were treated 21 days after i.p. injection of the tumor-derived cell aggregates.

b Mice were bled 5 days after treatment, and the serum levels of IFN-γ determined by ELISA. Each value represents the serum level from a single mouse.

c Mice were bled 78 days after treatment and the serum levels of human immunoglobulin determined by ELISA. Each value represents the serum level from a single mouse.

The viability and function of B cells and plasma cells in the tumor bearing mice were addressed by assaying the sera for the presence of human Ig. We consistently observed high levels of human Ig in the sera of tumor bearing mice (Table 2). In one representative experiment, elevated levels of Ig were observed in mice 78 days post treatment in both IL-12 treated and control mice, and no consistent enhancement of the serum Ig levels has yet been seen in response to IL-12.

The presence of both functional tumor-associated T and B cells in the tumor xenograft bearing mice for >100 days provides an opportunity to investigate the possible role that these lymphocytes (and the biologically active factors they produce, i.e. cytokines and antibodies) play in the survival and metastasis of the tumor, and to determine whether it is possible to manipulate these lymphocytes so that they mount an effective anti-tumor response *in situ*.

## Discussion

Since the first report on the successful engraftment of human cells in C.B-17-scid mice [1], several thousand papers have been published on the use of these and other immunodeficient mice to engraft human malignant and non-malignant tissues in studies of human cancer, hematopoiesis, adaptive and innate immunity, infectious diseases, autoimmunity and regenerative medicine [2]. Mouse models have been used to study cancer cell growth and to preclinically evaluate the therapeutic efficacy of immune based and non-immune based treatment strategies for cancer. Limitations of these earlier models included a significant innate immune response of the recipient mice that limited the duration of the graft and made it difficult to interpret results of therapeutic efficacy studies. These xenograft models failed to recapitulate the sites and patterns of tumor growth and in most of the models co-engraftment of stromal cells with the tumor was not established [3].

We report here a humanized model that more closely parallels the patterns of tumor progression that are observed in ovarian cancer patients than does any other previously described model. Our model captures several important features of human ovarian cancer not seen in previous models. The intra-abdominal spreading along with the development of ascites is strikingly similar to what is observed in patients. The co-engraftment of tumor-associated fibroblasts and T and B lymphocytes that remain viable and functional for prolonged periods provides an opportunity to investigate the possible contribution of these non-malignant cells, and the biologically active factors they produce, to the growth and spread of the tumor cells.

The orthotopic engraftment and dissemination of the tumor and co-engraftment of tumor-associated immunocompetent cells with tumor stroma in this new humanized mouse model make it possible to study *in vivo* the interaction between lymphocytes and the tumor, lymphocytes and tumor-associated fibroblasts and fibroblasts and the tumor. It has previously been established that tumor stroma is critical for preventing or permitting the immunological destruction of cancer cells [20]. This work was later confirmed [21] and then extended showing that the tumor stroma leads to a T cell mediated eradication of established tumor [22]. Orimo *et al.* showed that fibroblasts derived from human invasive breast carcinomas significantly enhanced tumor growth in xenograft models [23]. Tumor-associated fibroblasts have been shown by others to enhance or suppress T cell function [24]–[26] and subsets of tumor-associated T cells are known to interact with tumor cells and reciprocally modulate each other [27]. Whether fibroblast or leukocytes present within human tumor microenvironments enhance and/or suppress tumors remains to be established [28], [29]. By depleting, functionally inhibiting or activating specific subsets of cells within the tumor-derived cell aggregates prior to inoculation in NSG mice the effect upon the engraftment and subsequent intra-abdominal spreading and metastasis into the extra-abdominal pleural space can be determined, thereby establishing which cells in the tumor microenvironment contribute to tumor arrest or enhancement of tumor progression.

Ovarian cancer is most often asymptomatic in its early stages as most patients have widespread disease at the time of diagnosis [30]. New insights into the pathogenesis and origin of serous ovarian cancer may ultimately lead to an earlier diagnosis of this disease. Increasing evidence has indicated that most or all high-grade serous ovarian carcinomas originate in fallopian tubes [31], [32]. Elevated serum levels of CA125 (a tumor marker protein) are found in >90% of patients with advanced stage ovarian cancer, but comparatively few patients with Stage I disease are CA125 positive. Similarly mice inoculated i.p. with tumor-derived cell aggregates show no clinical evidence of tumors for at least 80 days when elevated CA125 levels in the sera were first detected. The serum CA125 level then increased rapidly reaching levels exceeding 500 units/ml by day 100 post inoculation. At this time and beyond the mice exhibited a pathology consistent with Stage IIIc and IV of ovarian cancer, i.e. evidence of intra-abdominal spread into multiple organ sites, diaphragmatic implants, development of tumor ascites and extra-abdominal spread into the pleural space with metastases to the lung [7].

Thus, while CA125 levels are a reliable indicator of the presence and expansion of ovarian tumors in patients and in our humanized mouse model, it is not suitable for early tumor detection in either patients or in mice inoculated with the tumor-derived cell aggregates. However, serum levels of CA125 are currently used effectively to monitor ovarian cancer patients' response to chemotherapy and surgery [8]. This biomarker has also been used as a prognostic indicator for ovarian cancer patients. We expect that serum CA125 levels will also be a very useful quantifiable marker to monitor periodically and compare *in vivo* therapeutic effects in our humanized mouse model.

The enhanced ability of small aggregates of tumor and non-malignant tumor cells to establish, grow and spread within the peritoneal cavity (compared to solid non-disrupted pieces of tumor tissue) may depend upon the ability of small cell clusters to survive initially until a sufficient vascular supply is established to permit tumor expansion. The success of the engraftment of tumor-derived cell aggregates may also depend in part upon the contribution of biologically active factors produced by the non-malignant cells in the cell aggregates that produce tumor growth factors and stimulants to angiogenesis. Tumor masses most often occur first in the cranial areas of the peritoneal cavity near and within the omentum. The position of the tumor nodules as well as the enhanced growth of the cell aggregates and slow progression of the tumors to other organs may result from the initial attachment of the tumor cell aggregates to highly vascularized areas within the omentum called milky spots. These discrete areas were first recognized by Ranvier [33] and have been shown to be sites where tumors attach preferentially and proliferate following the i.p. injection of several different murine tumor cell lines and a human ovarian tumor cell line into mice [34], [35]. Others have suggested that the intraperitoneal spreading of the tumor in ovarian cancer patients may occur by the attachment of tumor cells to the milky spots [36] and omentectomies have been included as part of the therapeutic approach in these patients [37]–[39] in an attempt to prevent further spreading of the tumor.

The results presented here suggest that our humanized NSG mouse ovarian transplant model has the potential to serve as a useful preclinical tool with which to test the efficacy of chemotherapeutic strategies, immunotherapeutic strategies or combinations of both. One of the attractions of this model is that it makes it possible to test strategies that target tumor-associated T cells and thereby initiate an anti-tumor response. One possible use of the model would be to test the ability of a cytokine such as IL-12 to initiate systemic anti-tumor responses. While the single treatment of tumor bearing mice 21 days post tumor inoculation did not result in a significant inhibition of tumor progression, future studies will assess the effect of multiple treatments with IL-12 or IL-12 in combination with other cytokines and chemotherapy. This type of an *in situ* strategy of cancer vaccination has already been demonstrated to be efficacious in several mouse tumor models [40]–[42].

The humanized mouse model described here has focused solely upon the engraftment of epithelial ovarian cancer tissues. Whether or not this approach could be used effectively to engraft and study other tumors that originate in the peritoneal cavity is currently being investigated. Other orthotopic xenograft models of intraperitoneal tumors have been reported [43], [44], but none have included the co-engraftment of the non-malignant tumor stroma or have recapitulated the patterns of growth and metastasis seen in patients.

A key factor in the success of the engraftment and long-term survival of human tumor xenografts has been the use of the NSG mice [5]. These mice have also been used most effectively to study the engraftment, growth and spontaneous metastasis of human tumor stem cells and have been shown to be significantly more efficient in the detection of tumorigenic cells [45], [46]. These severely immunodeficient mice will continue to be critical in establishing xenografts that recapitulate the growth and spreading patterns of tumors observed in patients and have the potential to serve as reliable preclinical models to evaluate cancer therapies.

## Materials and Methods

### Ethics Statement

Tumor tissues were obtained with an IRB-approved protocol covered under Human Subject Assurance Number 00008824 assigned through the UB Health Sciences Institutional Review Board accredited by the Association for the Accreditation of Human Research Protection Programs. All animal work was conducted with an Institutional Animal Care and Use Committee (IACUC) approval covered under Animal Welfare Assurance Number A3354-01 accredited through the Association for Assessment and Accreditation of Laboratory Animal Care.

### Tumor samples

The Roswell Park Cancer Institute Tissue Procurement Facility provided the human primary and metastatic ovarian solid tumor tissue and ovarian ascites fluid. All samples were provided under sterile conditions using Institute Review Board approved protocols.

### Processing of tumor

Tumor tissue was mechanically disrupted using a Teflon policeman to generate clusters or aggregates of cells that passed through a size 50 stainless steel wire mesh. The resulting tumor-derived cell aggregates were washed once in RPMI 1640 and resuspended in PBS, pH 7.2. Aggregates of cells in size from approximately 200–300 µm in diameter and included tumor cells, fibroblasts, endothelial cells and lymphocytes.

### Preparation and loading of IL-12 liposomes

Large multilamellar liposomes were prepared by rehydrating the lipid film of appropriate molar ratios of distearoyl phosphatidylcholine (DSPC), dimyristoyl phosphatidylglycerol (DMPG) and cholesterol (CHOL) (DSPC∶DMPG∶CHOL; 80∶20∶25) with phosphate buffer containing recombinant human IL-12 at 45°C. The spontaneous loading of a large complex molecule such as IL-12 is accomplished by a mild denaturation of IL-12 to partially and reversibly unfold the protein to expose hydrophobic domains resulting in the intercalation of the cytokine within or between the lipid bilayers of the liposomes [19]. This technique called triggered loading results in an optimal loading of the IL-12 which retains its biological activity. After rehydration lipids are gently swirled and incubated at 45° for 10 minutes, then 15 minutes at 4°C for 3 cycles as described in Simpson-Abelson *et al.* [19].

### Tumor implantation into mice

Suspensions of tumor-derived cell aggregates were injected i.p. into NSG mice. All NSG mice raised in a high barrier research colony at The Jackson Laboratory, were housed in specific pathogen-free conditions at SUNY at Buffalo. Each mouse received between 100 to 300 mg of the cell aggregates in a volume of 1.0 ml. All animal experiments were approved by the Institute Animal Care and Use Committee at SUNY at Buffalo.

### Treatment of mice with recombinant human IL-12

Wyeth Laboratory provided the recombinant human IL-12 that was incorporated into liposomes as described above. Mice bearing the human ovarian tumor xenograft were randomly divided into control and treatment groups. Mice were treated with a) empty liposomes, b) IL-12 containing liposomes (50 µg/mouse) – once or multiple times or c) left untreated. The dose of IL-12 was determined based upon the calculated loading efficiency for each liposome preparation. All of the cytokine was associated with the liposome as the free IL-12 was removed from the liposome preparations prior to injection.

### Mouse serum or plasma collection

Blood samples were collected via a retro-orbital sinus bleed. Clotted blood was centrifuged at 1000×g for 20 minutes and sera harvested and stored at −20°C until use.

### Human IFN-γ ELISA

A sandwich ELISA for the detection of human IFN-γ in mouse serum was performed as previously described [24]. Briefly 96 well plates were coated using a monoclonal antibody to IFN-γ (M-700-a; Endogen). Dilutions of mouse sera were added to the plate with a biotinylated monoclonal anti-human IFN-γ (M-701-B; Endogen). Positive binding was detected using strepavidin-conjugated HRP (Sigma–Aldrich), peroxide and 3,3′,5,5′-tetramethylbenzidine (SureBlue from KPL). Results were measured on a Bio-Tek Instruments automated microplate reader at OD_450–540_ and analyzed by comparison with a recombinant IFN-γ standard using SigmaPlot software.

### Human IgG Immunoassay

The ELISA for the detection of human Ig (huIg) in mouse serum was reported previously [5]. Briefly, 96 well plates were coated using rabbit anti-human Ig (Accurate Chemical, Westbury, NY) and incubated overnight at 4°C. The plates were washed. Bound huIg was detected using rabbit anti-human Ig-HRP, and a tetramethylbenzidene (TMB) peroxidase substrate (SureBlue from KPL, Gaithersburg, MD). Results were measured on a BioTek microplate reader at OD_450–540_ and analyzed with a human Ig standard (from Accurate Chemical, Westbury, NY) using Sigma Plot software.

### CA125 Immunoassay

Levels of CA 125 were determined by analysis with the Bayer Immuno ITM CA-125 II assay (Bayer Corp, Pittsburgh, PA).

### Histology and immunohistochemistry

H&E staining was performed by the SUNY at Buffalo Histology Service Laboratory where fresh surgical sections of the original tumor, xenografts and mouse tissues were fixed in 10% neutral-buffered formalin and processed for paraffin embedding. Anti-human specific antibodies to the following markers were used for immunohistochemical staining of the xenografts and mouse tissues: anti-CD3 (Dako), anti huHLA-A Class I (Santa Cruz), anti-huCD45 (R&D Systems), anti-hu 138 (Dako) and anti-hu CD20 (ab Cam). Staining was performed as previously reported [5].
